# 24-hour movement behaviours in the early years, potential behavioural determinants and prospective associations with growth, motor and social–emotional development: the My Little Moves study protocol

**DOI:** 10.1136/bmjopen-2023-081836

**Published:** 2024-10-22

**Authors:** Teatske M Altenburg, Jessica S Gubbels, Jelle Arts, Annelinde Lettink, Sanne Veldman, Arnoud Verhoeff, Mai Chinapaw

**Affiliations:** 1 Department of Public and Occupational Health, Amsterdam UMC Location VUmc, Amsterdam, Noord-Holland, The Netherlands; 2 Health Behaviours and Chronic Diseases, Amsterdam Public Health Research Institute, Amsterdam, The Netherlands; 3 Department of Health Promotion, NUTRIM School of Nutrition and Translational Research in Metabolism, Maastricht University, Maastricht, The Netherlands; 4 Mulier Institute, Utrecht, The Netherlands; 5 Public Health Service Amsterdam, Sarphati Amsterdam, Amsterdam, The Netherlands; 6 Department of Sociology, University of Amsterdam, Amsterdam, The Netherlands

**Keywords:** public health, epidemiology, community child health

## Abstract

**Abstract:**

**Introduction:**

The early years are a critical period for establishing healthy 24-hour movement behaviours (physical activity, sedentary behaviour and sleep), yet studies examining prospective associations between all 24-hour movement behaviours and young children’s growth and development are lacking. The My Little Moves study aims to (1) examine the prospective association between 24-hour movement behaviours of young children (ie, 0–4 years) and their growth, motor and social–emotional development; and (2) explore potential determinants of young children’s 24-hour movement behaviours from an ecological perspective, to inform public health strategies aimed at promoting healthy behaviours and development.

**Methods and analysis:**

My Little Moves is a longitudinal observational cohort study, with data collection at baseline, and after 9 and 18 months follow-up. Data are collected in three subcohorts. In all subcohorts, 24-hour movement behaviours are assessed by parent-report. Additionally in subcohort 1, data on potential determinants are collected by parental questionnaires, including child, parental and environmental factors. In subcohort 2, social–emotional development is assessed using the Dutch version of the Bayley Scales of Infant and Toddler Development-third edition (Bayley-III-NL) Social Emotional Scale. In subcohort 3, data on height and weight, gross motor development, using the Bayley-III-NL Gross Motor Scale, and 7 consecutive days of 24-hour accelerometer data are collected. Hybrid model analyses are used to assess the prospective associations of 24-hour movement behaviours with young children’s growth and development. Potential determinants of young children’s 24-hour movement behaviours are explored using regression analysis.

**Ethics and dissemination:**

The Medical Ethics Committee of the VU University Medical Center approved the protocol for the My Little Moves study (2022.0020). The results of this study will be disseminated through the network of all authors, to inform public health strategies for promoting healthy 24-hour movement behaviours and contribute to the evidence-base of recommendations for ideal 24-hour movement behaviours in young children.

**Strengths and limitations of this study**

- Collecting a large variety of complimentary data including both parent-reported and accelerometer data on young children’s 24-hour movement behaviours, their gross motor development, social–emotional development and potential determinants of their 24-hour movement behaviour is a an important strength of the My Little Moves study.
- A limitation is the design of the My Little Moves study with data collection in three subcohorts, each addressing a specific aspect of the research aim, which prevents analyses of all longitudinal associations between 24-hour movement behaviours, health and developmental outcomes and determinants in the same sample.
- Another limitation is that the My Little Moves app could not be validated before the start of the data collection, due to a delay in the development of the app.
- The sophisticated data analysis is another strength of this study, accounting for the alternation and accumulation of accelerometer-based 24-hour movement behaviours, and using both hip-based and wrist-based accelerometers data therewith capturing the child’s movement of the arms and the body centre of gravity.

## Introduction

 The benefits of physical activity for physical, social and cognitive health in children and adolescents is well known.^1 2^ As low levels of sedentary and screen time, and optimal sleep duration additionally have been associated with health benefits among children and adolescents,^13^,^5^ it is now recognised that all so-called 24-hour movement behaviours are important for optimal health and development.^4 6^ To address this, a number of countries have developed and released 24-hour movement guidelines for older children and youth (5–17 years).^7 8^ Using these guidelines, a recent meta-analysis demonstrated that about one in five young people (aged 3–18 years) from 23 countries do not meet any of the three 24-hour movement guidelines.^9^

The early years (ie, <5 years) are a critical period for establishing healthy 24-hour movement behaviours later in life,^10 11^ yet only a few studies have examined the relationship between all 24-hour movement behaviours and young children’s growth and development, of which none included children <3 years.^12^ The limited number of studies in the early years and therewith lack of evidence on optimal 24-hour movement behaviours is likely due to challenges in the assessment and analysis of 24-hour movement behaviours at this age. Accurate assessment of 24-hour movement behaviours in young children is challenged by the rapid and high variability in motor development.^13 14^ As a result, assessment needs to be adapted to the child’s developmental stage.^15 16^ For example, proxy-report questionnaires need to be tailored to fit the specific behaviours of babies, infants, toddlers and preschoolers (eg, tummy time, crawling, walking), and enable reporting of the intermittent and unstructured pattern of their movement behaviours. For accelerometer-based assessments, accelerometer placement needs to be adjusted to fit the child’s developmental stage (eg, tummy time, crawling, walking). In addition, specific algorithms are required to identify specific movements that do not reflect physical activity, such as ‘being carried’ by an adult. Similar to other age groups, the analysis of all 24-hour movement behaviours combined is challenged by the codependency of the 24-hour movement behaviours, causing collinearity problems,^17^ and the alternation and accumulation of these behaviours in a 24-hour and 7 days period, which may have a different influence on children’s growth and development than the total volume of these behaviours.^18^

To stimulate young children’s healthy growth and development, knowledge on determinants of healthy 24-hour movement behaviours is required. However, recent reviews summarising the evidence on potential determinants of physical activity, screen behaviours and sleep generally concluded that there is either insufficient or inconsistent evidence for most included determinants.^19^,^22^ Main reasons for this include a lack of valid and reliable measurement instruments, both for assessing 24-hour movement behaviours and potential determinants; and the focus on simple linear relationships instead of taking a more comprehensive or multifactorial approach, considering both individual and environmental factors as well as their interactions.^23^ Further, there is a lack of studies in the youngest age group (ie, <2 years).

The My Little Moves study addresses the above-mentioned challenges and gaps in knowledge by (1) developing measurement instruments (parent-reported data) and protocols (accelerometer-based data) to assess young children’s 24-hour movement behaviours tailored to the child’s developmental stage; (2) examining the prospective association between 24-hour movement behaviours and young children’s growth, motor and social–emotional development; and (3) examining potential determinants of young children’s 24-hour movement behaviours from an ecological perspective and taking a multifactorial analysis approach. This paper describes the protocol for data collection within the My Little Moves study. The development and content validity of the parent-report measurement instrument is described elsewhere.^24^

## Methods

### Study design and participants

The My Little Moves study is a longitudinal observational cohort study that is conducted in the Netherlands, with data collection at three time points: at baseline (between April 2022 and August 2023), and after 9 and 18 months. To limit the burden for the participating children and their parents, children were included in one of three subcohorts, each addressing a specific aspect of the research aim: subcohort 1 examines potential determinants of young children’s 24-hour movement behaviours; subcohort 2 examines the prospective association between young children’s 24-hour movement behaviours and their social–emotional development; and subcohort 3 examines the prospective association of young children’s 24-hour movement behaviours and their growth and gross motor development. We aimed to include 600 children (50% girls), stratified by cohort and age group to cover the entire 0–4 age range at baseline: (1) 0–6 months; (2) 6–12 months; (3) 12–18 months; (4) 18–24 months; (5) 24–36 months and (6) 36–48 months. Taking into account a potential drop-out of 33%, we aimed to include 800 children at baseline (ie, 270 children per cohort with 45 children per age group). Children’s participation in follow-up measurements continues until a maximum age of 48 months. As a result, the number of follow-up assessments varies per child.

Children were eligible if they were apparently healthy, which was defined as typically developing children born term or moderate to late preterm (>32 weeks) and without parent-reported known developmental disorders or medical diagnoses that might influence their 24-hour movement behaviours or development. Additionally, parents needed to have basic Dutch language reading skills and own a smartphone or tablet device for data collection on 24-hour movement behaviours. Informed consent was obtained from parents before inclusion of their child. The Medical Ethics Committee of the VU University Medical Center approved the protocol for the My Little Moves study (nr. 2022.0020).

### Recruitment

Originally, we planned to recruit children—via their parents—through the dynamic Sarphati cohort (https://www.sarphaticohort.nl/en/) and early childhood education and care (ECEC) services, within the city of Amsterdam, the Netherlands. However, to speed up recruitment, from January 2023 onwards children were additionally recruited through other community organisations and public places in Amsterdam, for example, organisations delivering sports and physical activity for young children, public libraries and playgrounds. Additionally, from January 2023, children were recruited through ECEC services, youth healthcare services and community organisations outside Amsterdam. Parents received an information letter containing a link to the My Little Moves project website (www.amsterdamumc.org/en/research/institutes/amsterdam-public-health/highlights/my-little-moves.htm). This website contained all information on the project, an informative video and a link for obtaining online informed consent using ‘Survalyzer’ software. Alternatively, parents could print and return a paper format of the informed consent form. Parents could also scan the QR code on a flyer that linked them to the online information letter and informed consent. All information (eg, information letter, video, informed consent form) was adapted to the specific subcohort. At the start, waves of email invitations (Sarphati cohort) and organisations (ECEC and youth healthcare services, community organisations) were randomly allocated to subcohorts 1 and 2. At a later stage, allocation was targeted to balance the sample size across subcohorts. For subcohort 3, the data collection is more intensive (requiring in-person contact with the child) and time consuming (ie, physical measurements and wearing accelerometers). Therefore, we recruited children for subcohort 3 through organisations that could support recruitment through face-to-face contact and offer a space for conducting the measurements, that is, ECEC and youth healthcare services.

#### Sarphati cohort

Within the Sarphati cohort, research is conducted on the growth and development of Amsterdam children. This concerns children receiving services from Youth Healthcare at the Public Health Service of Amsterdam (GGD Amsterdam) and the Parent and Child Teams of the Amsterdam Health Center Foundation. The Sarphati cohort includes a multiethnic, apparently healthy sample of young children aged 0–48 months, living in Amsterdam. The Sarphati cohort contains sociodemographic data (eg, age, sex and ethnicity of children and parents, parental education level), health and developmental data (eg, height and weight of both children and their parents), behavioural data (eg, parent-reported sleep, dietary intake, screen use of their child) and environmental data (residence and living environment) available for research. Detailed information on the Sarphati cohort is published at http://www.sarphaticohort.nl/en. All children in the Sarphati cohort aged between 4.5 and 48 months received an invitation and information letter to participate in the My Little Moves study by email. Parents and children in the Sarphati cohort were recruited for participation in subcohort 1 or 2.

#### ECEC and youth healthcare services

ECEC and youth healthcare services were recruited using existing networks of the My Little Moves consortium and a list of ECEC services (eg, early childhood day care centres and preschools), created by the researchers. Managers of ECEC and youth healthcare services were approached for study participation by phone or email and—in case of agreement—were asked to send the information letter to parents through email, newsletters and/or flyers by professionals working at these services. If possible, researchers visited the locations of ECEC and youth healthcare services to recruit parents and children. Parents and children approached via ECEC and youth healthcare services were recruited for participation in subcohort 1, 2 or 3.

#### Other community organisations and public spaces in Amsterdam

Community organisations (eg, public libraries, organisations delivering sports and physical activities for young children) were approached with the request to allow the researchers to recruit parents who visit the organisation with their children in-person, by handing information letters. Researchers also approached parents visiting public spaces (eg, playgrounds) with their children handing out information letters. Parents and children approached via other community organisations and public spaces were recruited for participation in subcohort 1 or 2.

### Data collection

#### Procedures

Baseline measurements were collected from June 2022 until August 2023. Parents agreeing to participate provided the birth date and gender of their child through the informed consent forms. In subcohort 1, informed consent was obtained for 219 children of which 214 children (20.5±12.4 months, 53% girls) were eligible. In subcohort 2, informed consent was obtained for 202 children of which 197 children (12.3±13.0 months, 46.7% girls) were eligible. In subcohort 3, informed consent was obtained for 87 children of which 84 children (19.1±11.8 months, 43% girls) were eligible for inclusion.

Data on 24-hour movement behaviours are collected by parents in all subcohorts using the My Little Moves app, and additionally by accelerometers in children included in subcohort 3. Data on potential determinants of 24-hour movement behaviours are collected in subcohort 1, data on social–emotional development in subcohort 2 and data on growth and motor development in subcohort 3. After receiving informed consent, parents receive detailed information and links (through e-mail or the data management platform Castor EDC) to complete data collection for the subcohort their child is enrolled in. If preferred, parents receive a paper format of the questionnaire. For cohort 3, in consultation with ECEC professionals and/or parents, researchers schedule a time/day to assess children’s growth and motor development either at the ECEC centre or at their home. After assessing children’s growth and motor development, children were equipped with two accelerometers. All data is collected by trained researchers and according to standardised protocols. Table 1 provides an overview of all study variables and measurement instruments, with reference to the corresponding cohorts.

**Table 1:** Overview of collected data, with reference to the corresponding subcohorts

Study construct	Measurement instrument	Subcohort
24-hour movement behaviours—proxy-report Physical activity, sedentary behaviour and sleep Activity categories of the My Little Moves app*	My Little Moves app, completed by parents/caregivers	All subcohorts
24-hour movement behaviours—device-based Summary measures, including average acceleration, 25th percentile of acceleration	Axivity AX3 devices, placed on children’s right hip and left wrist	Subcohort 3
Potential determinants: Child factors, including age and gender Parental factors, including attitudes and practices, sociodemographic characteristics, parents’ own physical activity and sedentary behaviour Environmental factors, including home environment, neighbourhood environment, childhood day-care centre and preschool environment†	Customised questionnaire based on items of previously developed validated questionnaires, including PPAPP,^27 28^ Parenting SOS questionnaire,^29^ SUNRISE questionnaire,^11^ BISQ-R short form,^31^ IPAQ-SF,^32 33^ EPAO instrument^23 34^	Subcohort 1
Social–emotional development	Bayley-III-NL Social-Emotional Scale^37 38^	Subcohort 2
Gross motor development	Bayley-III-NL Gross Motor Scale^37 38^	Subcohort 3
Growth BMI-Z scores	Height: portable stadiometer or length board‡Weight: calibrated electronic (baby) scale	Subcohort 3

* Activity categories within My Little Moves app include: personal care, active transport, passive transport, playing, screen use, sitting/lying calmly and sleep.^24^

† Environment of childhood day-care centres and preschools (ECECearly childhood education and care services) only assessed in case of recruitment via these organisations.

‡ Depending on the child’s ability to stand.

Bayley-III-NL, Dutch version of the Bayley Scales of Infant and Toddler Development-third edition; BISQ-R, Brief Infant Sleep Questionnaire Revisited; BMI, body mass index; EPAO, Environment and Policy Assessment and Observations; IPAQ-SF, International Physical Activity Questionnaire Short Form; PPAPP, Preschooler Physical Activity Parenting Practices

In case parents do not complete the requested My Little Moves app or questionnaires for data collection, they receive reminders 1 week and 3 weeks after receiving the original email with the request. For subcohort 3, when parents do not report any of their child’s activities in the My Little Moves app after 3 days, they receive a reminder to assure alignment between app and accelerometer data.

#### 24-Hour movement behaviours: parent-report (subcohorts 1, 2 and 3)

Parents receive an email including a request to download the My Little Moves app and detailed information on how to complete the app. Parents are asked to report the activities of their child for 7 consecutive days in the My Little Moves app, which was developed with the input of parents and professionals working with young children (see online supplemental file 1 for screenshots of the My Little Moves app). The app has a time-use diary format through which parents report all activities of their child, using the following activity categories: personal care, eating/drinking, active transport, passive transport, playing, screen use, sitting/lying calmly and sleep. Additionally, depending on the chosen category and age of the child, parents respond to follow-up questions regarding intensity (eg, for playing: active, calm), posture (eg, lying on tummy, being carried, standing with support), location (eg, indoor, outdoor), who were present (eg, one or more children, one or more adults) and type of device (for screen use). Reporting activities in the app takes about 10–30 min per day. Before filling in the timeline, on each measurement point, parents provide information on the age of their child (answering categories: 0–6 months, 6–12 months, 1–2 years, 2–3 years and 3–4 years) and the motor ‘milestones’ their child has reached and at which age these were achieved. These ‘milestones’ include roll over from back to belly, roll over from belly to back, sit without support for 5 s, crawl for 1.5 m, stand without support for 3 s, walk three steps without support. The content of the app (ie, activity categories and follow-up questions) is tailored to the age and developmental stage of the child, based on the selected age group and meeting the above-described ‘milestones’.

In a content validity study, parents (n=14) and researchers (n=6) considered the My Little Moves app comprehensive, user-friendly, comprehensible and feasible to use.^24^ Data from the My Little Moves app are downloaded by the software developer, and subsequently transferred to the research team using a safe and secure web-based system (ZendTo). Children’s total time spent in each of the activity categories and total time spent in physical activity, sedentary behaviour and sleep are calculated.

#### 24-Hour movement behaviours: accelerometers (subcohort 3)

**Figure 1:**
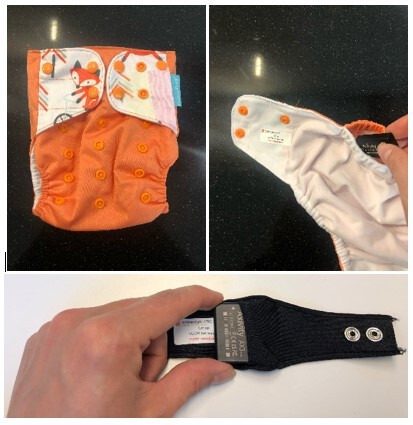
Placement of Axivity AX3 monitors in diaper pants and wristband.

Data on body movement of children are collected using Axivity AX3 devices (Axivity Ltd, UK). Axivity AX3 devices are small (23×32.5×7.6 mm) and lightweight (11 g) devices that capture data on body movement (raw acceleration data, expressed in gravitational units (g)), or lack thereof, over extended periods of time. One Axivity AX3 device is placed on the child’s right hip (incorporated in diaper pants that can easily be worn over the regular diaper for younger children and in a short tight pants for older children to be worn over their underwear) and one device is placed on the child’s left wrist (using wristbands^25^). Device is worn for 8 consecutive days, except during showering, bathing or other water activities. Parents receive detailed instructions on the procedures regarding placement of the devices. Figure 1 presents the placements of the Axivity AX3 devices in diaper pants and wristbands.

Axivity AX3 data are processed using R package GGIR V.2.9-0,^26^ including detection of sustained abnormally high values, detection of non-wear time and calculation of acceleration in gravitational units averaged over (to be specified) epoch lengths.

#### Potential determinants of 24-hour movement behaviours (subcohort 1)

Potential determinants of 24-hour movement behaviours include child, parental and environmental factors assessed by parent-report. Child factors include age and gender, and are collected through the informed consent form. Questions on parental and environmental factors are adapted from items from existing validated questionnaires, including the Preschooler Physical Activity Parenting Practices^27 28^ (PPAPP; Dutch translation), Parenting SOS questionnaire,^29^ SUNRISE questionnaire,^30^ Brief Infant Sleep Questionnaire Revisited (BISQ-R) short form,^31^ International Physical Activity Questionnaire (IPAQ) short form^32 33^ and Environment and Policy Assessment and Observation (EPAO) instrument.^34^ Parental factors include 63 items on attitudes and parenting practices regarding their child’s physical activity (38 items), screen behaviour (13 items), sedentary behaviour (4 items) and sleep (8 items), including both factors on encouraging and discouraging these behaviours. Additionally, the questionnaire includes 15 items on parental sociodemographic characteristics (eg, age, sex, country of birth, educational level), 4 items on parents’ own physical activity (3 items; time spent in light, moderate and vigorous activities) and sedentary behaviour (1 item; time spent in sedentary behaviours). Environmental factors include 3 items on the home environment (eg, presence of outdoor space), and 12 items on the neighbourhood (9 items on physical environmental factors, eg, presence of a park, green space or playground; 3 items on neighbourhood safety). The questionnaire takes about 30 min to complete. Online supplemental file 2 presents all questionnaire items included in the My Little Moves determinants questionnaire, including references to original sources and information on validity and reliability. Questionnaire data are transferred from Castor EDC to SPSS to be analysed on constructs related to parenting practices, parental behaviours, children’s sleep and the living environment.

For all children included in subcohort 3 recruited via ECEC services, we approached the managers for approval to assess the ECEC physical and policy environment. After approval, one research assistant assessed the ECEC environment an policy during a day long visit, using a Dutch translation of the Environment and Policy Assessment and Observation (EPAO) instrument. This instrument assesses the provision, practices and policies around physical activity.^23 35 36^ The instrument consists of five subscales: activities during the morning (37 items), nap time/sleep (7 items), activities in the afternoon (38 items), physical activities (14 items) and equipment and environment (14 items). Observation data ares entered in an Excel form and subsequently converted to SPSS and analysed on constructs related to opportunities, equipment and natural environment, social environment and policies related to physical activity.

#### Social–emotional (subcohort 2) and gross motor (subcohort 3) development

Social–emotional and gross motor development are assessed using the Dutch version of the Bayley Scales of Infant and Toddler Development-third edition (Bayley-III-NL) Motor Scale and Social Emotional Scale.^37 38^ The Bayley-III-NL Social-Emotional Scale is a parent-report questionnaire to assess functional emotional milestones of young children. The scale contains 11 items for the youngest age group (0–3 months old) and items are added as the child gets older with a maximum of 35 items. All items are scored on a 5-point scale (0=can’t tell; 1=none of the time; 2=some of the time; 3=half of the time; 4=most of the time; and 5=all of the time). It takes approximately 10–15 min to complete all required items. Examples of items included are ‘Child pays attention to things around them in a calm and pleasant way, including colourful and brightly coloured objects’, ‘Child imitates many of your sounds, words or actions while you play with them’. Previous studies showed sufficient interitem reliability (λ−2=0.92), inter-rater reliability (r=0.59 between father and mother) and discriminant validity (p<0.001 when compared with a clinical sample).^38^ Data are transferred from Castor EDC to SPSS, and subsequently a sum score is calculated for social–emotional development, which is then transformed into norm-referenced scores (eg, index score) using Pearson’s Q-Global (Pearson Benelux B.V., Amsterdam, the Netherlands).

The Bayley-III-NL Motor Scale is an individually administered test that consists of two subtests: fine motor skills (66 items), and gross motor skills (72 items). In our study, only gross motor skills are assessed to limit the burden of the participants. Examples of items included in the test are sitting unsupported for at least 5 s, crawling on hands and knees for at least 1.5 m and jumping forwards for at least 60 cm. The age of the child determines the entry point of the test. If a child scores a 0 on any of the first five test items, the test is continued at the entry point associated with the previous age level. The test is discontinued if the child is not able to perform five consecutive test items correctly. To calculate the total score, the number of items for which a child tests positive (score 1) are added to the number of items that were not administered, prior to the lowest administered item. Administration of this subtest takes about 30 min per child, if preferred, in the presence of (one of) the parents. Previous studies showed sufficient interitem reliability (λ−2=0.88), test–retest reliability (r=0.75 and 0.60 between same and different test leaders, respectively), convergent validity (r=0.44 when compared with the BSID-II-NL Motor Scale) and discriminant validity (p<0.001 when compared with a clinical sample).^38^ The gross motor skill sum score is calculated, entered in Castor EDC and transferred to SPSS, and then transformed into norm-referenced scores using Pearson’s Q-Global (Pearson Benelux B.V., Amsterdam, the Netherlands).

#### Growth (subcohort 3)

Height and weight (used to calculate BMI Z-score) were measured at the location of participating ECEC and youth healthcare services or in their own home, with shoes and outer clothing removed and using standardised age-adjusted WHO protocols.^39^ In short, height (cm) is measured to the nearest 0.1 cm using a portable stadiometer (Marsden HM-250P) or a length board (Seca 417) depending on the child’s ability to stand. Weight (kg) is measured to the nearest 0.1 kg using a calibrated electronic (baby) scale (Seca 354). Measurements for height and weight are conducted twice. In case the second measurement differs more than 0.5 cm or 0.2 kg, respectively, from the first, a third measurement is conducted. Measurements take about 5 min. The average of two—or three—measurements is included in the analysis.

### Statistical analyses

Data are presented as means±SD for continuous variables, or median (IQR) in case data are not normally distributed, and as percentages for categorical variables. All statistics are conducted in SPSS (V.28) and R (V.44.2.1). The level of significance is set at p<0.05.

By using the Spearman-Brown prophecy formula, inputting single-day intraclass correlation coefficients, the minimum number of days and hours of registration time required to achieve a reliability of 0.70 is determined, both for parent-reported and accelerometer data on 24-hour movement behaviours.^40 41^ The construct validity of the My Little Moves app and accelerometers is examined by fitting general linear mixed-effects models to test for differences in accelerometer-derived acceleration across app-derived activity categories as well as physical activity, sedentary behaviour and sleep.

Hybrid mixed-model analyses are used to assess the prospective associations of 24-hour movement behaviours (based on summary measures of the My Little Moves app data and/or accelerometer data) with young children’s growth, motor and social–emotional development, respectively. Using a hybrid model, we include both the individual mean score and the deviation score around the individual mean as independent variables, which enables us to disentangle the between-subjects and the within-subjects part of the prospective associations.^42^ The codependency of the 24-hour movement behaviours is addressed using compositional data analysis.^17^ All analyses are adjusted for children’s age and gender.

Additionally, latent profile analysis is applied to the My Little Moves app data to identify 24-hour movement behaviour profiles and sequence mapping is applied to accelerometer data to explore the alternation and accumulation of 24-hour movement behaviours.^18^

Potential determinants of young children’s 24-hour movement behaviours (based on summary measures of the My Little Moves app data) are explored using regression analysis, including the child, parental and environmental factors as well as two-way interactions between these factors. First, 5–10 potential determinants and two-way interactions between these potential determinants are selected, based on relevant literature and theories. Next, associations of the selected potential determinants and two-way interactions with young children’s 24-hour movement behaviours are explored using regression analysis.

## Discussion

The My Little Moves study contributes to the evidence-building of recommendations for ideal 24-hour movement behaviours in young children, by examining the longitudinal associations of young children’s 24-hour movement behaviours (based on parent-report and/or accelerometer data) and their growth and motor and social–emotional development. Through sophisticated analyses of accelerometer-based 24-hour movement data, My Little Moves study delivers detailed information on young children’s 24-hour movement patterns, in which the accumulation and alternation of physical activity, sedentary behaviour and sleep is considered. As currently accurate proxy-report tools for assessing young children’s 24-hour movement behaviours are lacking,^15^ a parent-report tool is developed with the involvement of parents and key stakeholders.^24^ Through the My Little Moves app, this study addresses the challenge of collecting proxy-report data on 24-hour movement behaviours in young children.

By exploring potential determinants of young children’s 24-hour movement behaviours, the My Little Moves study additionally contributes to guiding public health strategies for promoting healthy 24-hour movement behaviours in the early years. The My Little Moves study addresses the current gap in knowledge regarding determinants of 24-hour movement behaviours,^19^,^21^ by exploring potential determinants from an ecological perspective taking into account interactions between potential determinants.

Strengths of My Little Moves include the collection of a large variety of complimentary data: both parent-reported and accelerometer data on young children’s 24-hour movement behaviours, their gross motor development, social–emotional development and potential determinants of their 24-hour movement behaviours. Additional strengths are the sophisticated analyses of the alternation and accumulation of accelerometer-based 24-hour movement behaviours, and using both hip-based and wrist-based accelerometers data therewith capturing the child’s movement of the arms and the body centre of gravity.

Limitations of the My Little Moves study include the design of the study with data collection in three subcohorts, each addressing a specific aspect of the research aim, to limit the burden of participating children and parents. This prevents us from examining all longitudinal associations between 24-hour movement behaviours, health and developmental outcomes and determinants in the same sample. An additional limitation is that, due to a delay in the development of the My Little Moves app, the app could not be validated before the start of the data collection. Finally, at the time of recruitment the Sarphati cohort (used for the recruitment of children for subcohorts 1 and 2) mainly included children from parents with a higher educational level, which limits the generalisability of the results with respect to determinants of 24-hour movement behaviours and longitudinal associations with social-–emotional development.

In conclusion, the My Little Moves study addresses important challenges and gaps in knowledge around healthy 24-hour movement behaviours in early childhood. This is important to stimulate children’s healthy growth and development and reduce lifestyle-related disease risk later in life.

### Patient and public involvement

The My Little Moves study is initiated by a consortium consisting of (academic) researchers and professionals working in the field of public health, knowledge dissemination, physical activity promotion and childcare. The My Little Moves Advisory board—consisting of academic researchers, professionals experienced in young children’s movement (eg, physiotherapist), youth healthcare professionals, ECEC professionals and parents—is consulted multiple times to provide advice on app development, study design, participant recruitment and selection of potential determinants. The My Little Moves app is developed in collaboration with parents of and professionals working with young children.

## Ethics and dissemination

The Medical Ethics Committee of the VU University Medical Center approved this study in the Netherlands (nr. 2022.0020). The results of this study will be disseminated through the diverse networks of all authors and participants.

## supplementary material

10.1136/bmjopen-2023-081836 online supplemental file 1

10.1136/bmjopen-2023-081836 online supplemental file 2
