# Supplementary material for: 24-hour movement behaviours in the early years, potential behavioural determinants and prospective associations with growth, motor and social–emotional development: the My Little Moves study protocol
Source: BMJ Open. 2024 Oct 22;14(10):e081836. doi: 10.1136/bmjopen-2023-081836 (PMC11499838; doi:10.1136/bmjopen-2023-081836)
Supplement: online supplemental file 2 [file bmjopen-14-10-s002.pdf]

Appendix 1. The My Little Moves questionnaire on determinants of 24-hour movement behaviours: questionnaire items, source and psychometric properties.

Questionnaire item	Response options	Item derived from	Reliability/Validity
**Socio-demographic characteristics**			
1.1: Who is answering the questionnaire?	Biological mother/Biological father/Non-biological parent or caregiver/Other	Sarphati Cohort questionnaire[1]	No validity/reliability assessments
1.2: In which country was the child's biological mother born?	Netherlands/Surinam/Netherlands Antilles/Turkey/Morocco/Other	Sarphati Cohort questionnaire	
1.3. In which country was the child's biological father born?	Netherlands/Surinam/Netherlands Antilles/Turkey/Morocco/Other	Sarphati Cohort questionnaire	
1.4: What is your date of birth?	Day/Month/Year	Sarphati Cohort questionnaire	
1.5: What is your gender?	Male/Female/Other/I don't want to say	Sarphati Cohort questionnaire	
1.6: What is your highest completed education?	No education/Primary education/ Lower secondary education/Upper secondary education/Post-secondary non-tertiary education/Other[2]	Sarphati Cohort questionnaire	
1.7: What is the highest completed education of your partner?	No education/Primary education/ Lower secondary education/Upper secondary education/Post-secondary non-tertiary education/ Other	Sarphati Cohort questionnaire	
1.8: What is the postal code of your home address?	Four digits		
1.9: Does your child go to: a daycare, a child minder, informal care such as grandparents/ family/ acquaintances?	Hours per week/Days per week	Sarphati Cohort questionnaire	
1.10: What is the number of people living in your household?	Number of adults; number of children	Sarphati Cohort questionnaire	
1.11: What are the ages of the children in your household?	Years/months	Sarphati Cohort questionnaire	
1.12: What is the height of the biological mother?	Cm	Sarphati Cohort questionnaire	
1.13: What is the height of the biological father?	Cm	Sarphati Cohort questionnaire	
1.14: What is the weight of the biological mother?	Kg	Sarphati Cohort questionnaire	
1.15: What is the weight of the biological father?	Kg	Sarphati Cohort questionnaire	

**Parenting practices – Child physical activity**			
How often do you…			
2.1: Set an example for your child by exercising in front of him/her?	5-point scale; never-always	Translated to Dutch from PPAPP	Dutch translation of PPAPP by Gubbels et al, 2016 (1)<br><br>Test-retest reliability and internal consistency scores from subscales in original PPAPP questionnaire by O'Connor et al, 2014 (2):<br><br>Parental engagement original subscale (based on items 2.1, 2.3, 2.6, 2.7, 2.9, 2.12-2.14, 2.16, 2.18, 2.19, 2.23-2.25, 2.27):<br>ICC (95% CI)=0.85 (0.75, 0.91)<br>IIC=0.38<br>Cronbach's α=0.90<br><br>Promote inactivity original subscale (based on item 2.15, 2.22, 2.34):<br>ICC (95% CI)=0.59 (0.38, 0.85)<br>IIC=0.26<br>Cronbach's α=0.50<br><br>Promote screen time original subscale (based on items
2.2: Tell your child that he/she is not (yet) able to do a sport or active game or is not (yet) good enough?	5-point scale; never-always + not applicable	Translated to Dutch and adapted from PPAPP	
2.3: Play active games with your child (e.g. ball game or running)?	5-point scale; never-always + not applicable	Dutch translation of PPAPP	
2.4: Allow your child to watch TV for long periods of time?	5-point scale; never-always	Dutch translation of PPAPP	
2.5: Allow your child to play a lot of videogames?	5-point scale; never-always + not applicable	Dutch translation of PPAPP	
2.6: Go on a walk with your child?	5-point scale; never-always + not applicable	Dutch translation of PPAPP	
2.7: Say positive things to motivate your child to be more active?	5-point scale; never-always	Dutch translation of PPAPP	
2.8: Tell your child he/she will get hurt if he/she plays actively?	5-point scale; never-always + not applicable	Dutch translation of PPAPP	
2.9: Play a sport or active game together as a family?	5-point scale; never-always + not applicable	Dutch translation of PPAPP	
2.10: Not let your child play actively for fear of him/her getting dirty?	5-point scale; never-always + not applicable	Dutch translation of PPAPP	
2.11: Discipline your child for being too active?	5-point scale; never-always + not applicable	Dutch translation of PPAPP	
2.12: Give your child choices about what kind of activity or movement he/she wants to do?	5-point scale; never-always + not applicable	Adapted from Dutch translation of PPAPP	
2.13: Allow your child to pick an active game to do together?	5-point scale; never-always + not applicable	Translated from PPAPP	
2.14: Dance with your child?	5-point scale; never-always + not applicable	Translated from PPAPP	

2.15: Carry your child because he/she does not want to walk or crawl?	5-point scale; never-always + not applicable	Adapted from Dutch translation of PPAPP	2.4, 2.5, 2.17):<br>ICC (95% CI)=0.62 (0.41, 0.77)<br>IIC=0.34<br>Cronbach's α=0.61<br><br>Psychological control original subscale (based om items 2.2, 2.8, 2.10, 2.11, 2.21):<br>ICC (95% CI)=0.85 (0.75, 0.91) IIC=0.26<br>Cronbach's α=0.59<br><br>Restriction for safety concerns original subscale (based on items 2.33, 2.35, 2.36):<br>ICC (95% CI)= 0.56 (0.33, 0.73)<br>IIC=0.53<br>Cronbach's α = 0.82<br><br>The test-retest reliability scores for remaining original individual items:<br>Item 2.18: ICC (95% CI)=0.62 (0.41, 0.76)<br>Item 2.29: ICC (95% CI)=0.57 (0.34, 0.74)
2.16: Play an exercise game or sport with your child (e.g. hide and seek, tag or football)?	5-point scale; never-always + not applicable	Adapted from Dutch translation of PPAPP	
2.17 Keep your child occupied by letting him/her watch TV?	5-point scale; never-always	Dutch translation of PPAPP	
1.18: Teach your child that being active is good for his/her health?	5-point scale; never-always + not applicable	Dutch translation of PPAPP	
2.19: Take your child to the park, playground, petting zoo or forest?	5-point scale; never-always	Adapted from Dutch translation of PPAPP	
2.20: Keep your child inside your home all day, regardless of the weather	5-point scale; never-always	Adapted from Dutch translation of PPAPP	
2.21: Reward your child for being still?	5-point scale; never-always + not applicable	Dutch translation of PPAPP	
2.22: put your child in a buggy/stroller if your child could have walked or crawled?	5-point scale; never-always + not applicable	Adapted from Dutch translation of PPAPP	
2.23: Teach your child new and different ways to be active?	5-point scale; never-always	Dutch translation of PPAPP	
2.24: Does your child participate in an organized exercise activity (e.g. baby swimming, gymnastics, soccer or toddler dance)?	5-point scale; never-always	Adapted from Dutch translation of PPAPP	
2.25: Find age appropriate games that get your child moving?	5-point scale; never-always	Adapted from Dutch translation of PPAPP	
2.26: Not let your child play outside because there is not enough space to play?	5-point scale; never-always + not applicable	Dutch translation of PPAPP	
2.27: Set time aside for active play?	5-point scale; never-always	Dutch translation of PPAPP	
2.28: Not let your child participate in an organized exercise activity (e.g. baby swimming, gymnastics, football or toddler ballet) because it is too expensive?	5-point scale; never-always	Adapted from Dutch translation of PPAPP	
2.29: Have outdoor toys available for your child (e.g. sandpit, ball or (balance) bike) ?	5-point scale; never-always	Adapted from Dutch translation of PPAPP	

2.30: Allow your child to help you with chores outside (e.g. gardening or sweeping, tidying up toys?	5-point scale; never-always + not applicable	Adapted from Dutch translation of PPAPP	
2.31: Suggest that your child play outside?	point scale; never-always + not applicable	Dutch translation of PPAPP	
2.32: Not have time to play outdoors with your child?	point scale; never-always + not applicable	Dutch translation of PPAPP	
2.33: Let your child go outside to play?	5-point scale; never-always + not applicable	Adapted from Dutch translation of PPAPP	
2.34: Drive your child, when it was easy to walk?	5-point scale; never-always + not applicable	Dutch translation of PPAPP	
2.35: Not let your child play outside because you are worried about traffic?	5-point scale; never-always + not applicable	Dutch translation of PPAPP	
2.36: Not let your child play outside because you are worried about safety?	5-point scale; never-always + not applicable	Adapted from Dutch translation of PPAPP	
2.37: Not let your child play outside because of the weather?	5-point scale; never-always + not applicable	Adapted from Dutch translation of PPAPP	
2.38: Keep your child occupied with quiet activities (such as quit games, puzzles or reading)?	5-point scale; never-always	Adapted from Dutch translation of PPAPP	
**Parenting practices – Child screen behaviour**			
3.1: How often do you use a screen device to educate your child?	5-point scale; never-multiple times per day	Translated and adapted from Parent/Caregiver questionnaire SUNRISE	Parent/Caregiver questionnaire SUNRISE by Okely et al, 2021 (3)<br><br>No validity/reliability assessments
3.2: How often do you use a screen device to calm down your child when he/she is upset?	5-point scale; never-multiple times per day	Translated and adapted from Parent/Caregiver questionnaire SUNRISE	No validity/reliability assessments
3.3: How often do you use a screen device to keep your child busy while you do something else?	5-point scale; never-multiple times per day	Translated and adapted from Parent/Caregiver questionnaire SUNRISE	No validity/reliability assessments

3.4: How often do you offer screens to your child as a reward for good behaviour?	5-point scale; never-multiple times per day	Translated and adapted from Parenting SOS study: Screen time parenting, subscale ‘use of screen time to reward/control child behavior’	Internal consistency scores from original subscales of the parenting questionnaire of the Parenting SOS study by Vaughn et al, 2013 (4):<br><br>Use of screen time to reward/control child behavior original subscale (4 items):<br>Cronbach’s α = 0.79<br>Factor loadings = 0.57–0.94<br><br>Limiting/monitoring of screen time original subscale (10 items):<br>Cronbach’s α = 0.79<br>Factor loadings = 0.53–0.82
3.5: How often do you take away screens to your child as a punishment for bad behaviour?	5-point scale; never-multiple times per day	Translated and adapted from Parenting SOS study: Screen time parenting, subscale ‘use of screen time to reward/control child behavior’	
3.6: How often do you use screens with your child present?	5-point scale; never-multiple times per day	Translated and adapted from Parent/Caregiver questionnaire SUNRISE	No validity/reliability assessments
3.7: How often does your child use screens in the two hours before bedtime?	5-point scale; never-multiple times per day	Translated and adapted from Parent/Caregiver questionnaire SUNRISE	No validity/reliability assessments
3.8: Do you have rules regarding how much time your child is allowed to use screens?	Yes/no	Translated and adapted from Parenting SOS study: Screen time parenting, subscale ‘Limiting/monitoring of screen time’	For validity/reliability of original subscale, see questionnaire items 3.4 and 3.5
3.9: Do you have rules regarding when your child is allowed to use screens?	Yes/no	Added	No validity/reliability assessments

3.10: Do you monitor the screen use of your child?	Yes/no	Translated and adapted from Parenting SOS study: Screen time parenting, subscale 'Limiting/monitoring of screen time'	For validity/reliability of original subscale, see questionnaire items 3.4 and 3.5
3.11: Are there screens in the living room?	Yes/no	Added	No validity/reliability assessments
3.12: Are there screens in the room where your child sleeps?	Yes/no	Translated and adapted from Parent/Caregiver questionnaire SUNRISE	No validity/reliability assessments
3.13: Does your child have a screen?	Yes/no	Added	No validity/reliability assessments
**Parenting practices – child sedentary behaviour**			
4.1: How often in the past week was your child restrained for more than one hour at a time (e.g. in a stroller, dining chair, car seat, on the back of a bike)	5-point scale; never-multiple times per day	Translated and adapted from Parent/Caregiver questionnaire SUNRISE	No validity/reliability assessments
4.2: How often in de past week was your child restrained (e.g. bouncer or dinging chair) so you can get things done (e.g. to cook, do the household, to shower/personal care)?	5-point scale; never-multiple times per day	Translated and adapted from Parenting SOS study: Screen time parenting, subscale 'Explicit modelling and enjoyment of screen time'	No validity/reliability assessments
4.3: How often in the past week was your child restrained (e.g. bouncer, dinging chair or bicycle seat) because you think it is safer?	5-point scale; never-multiple times per day	Added	No validity/reliability assessments
4.4: How often in the past week was your child restrained (e.g. bouncer or dinging chair) because your child is satisfied?	5-point scale; never-multiple times per day	Added	No validity/reliability assessments
**Child sleep questions**			

5.1: Does your child have a consistent bedtime?	Yes, bedtime does not vary by more than 30 minutes each day/No, bedtime can vary more than 30 minutes each day	Translated and adapted from Parent/Caregiver questionnaire SUNRISE	No validity/reliability assessments
5.2: Does your child have a consistent wake-up time?	Yes, this time does not vary by more than 30 minutes each day/No, this time can vary more than 30 minutes each day	Translated and adapted from Parent/Caregiver questionnaire SUNRISE	
5.3: In the past three days, has your child not got enough sleep?	Yes/No	Translated and adapted from Parent/Caregiver questionnaire SUNRISE	
5.3.1 (if 5.3=Yes): What were the reasons for this insufficient sleep (multiple options possible)?	Outside noise/indoor noise/too hot/too cold/too much Too much light coming in to the room/Other	Translated and adapted from Parent/Caregiver questionnaire SUNRISE	
5.4: How many people are sleeping in the same room as your child?	Only the participating child/The participating child and other children/The participating child and adults	Translated and adapted from Parent/Caregiver questionnaire SUNRISE	
5.4.1 (if 5.4=The participating child and other children): How many other children?	Number	Translated and adapted from Parent/Caregiver questionnaire SUNRISE	
5.4.2 (if 5.4=The participating child and other adults): How many other adults?	Number	Translated and adapted from Parent/Caregiver questionnaire SUNRISE	
5.5: Typically, how difficult is bedtime?	5-point scale; Very easy-Very difficult	Translated from BISQ-R short form	BISQ-R by Mindell et al, 2019 (7)<br><br>Test-retest reliability scores based on a Spanish version of the BISQ-R by Cassanello et al, 2018 (8):<br>r = 0.848, with a kappa value of 0.939 (95% CI= 0.858–1.00)
5.6: How well does your child usually sleep at night?	5-point scale; Very well-Very poorly	Translated from BISQ-R short form	
5.7: Do you consider your child's sleep a problem?	5-point scale; Not a problem at all-A serious problem	Translated from BISQ-R short form	
5.8: Does your child get night feeding?	Yes/No	Added	

5.8.1 (if 5.8=yes): How many times a night does your child get night feeding?	Number	Added	No validity/reliability assessments
**Parental physical activity and sedentary behaviour**			
6.1: During the last 7 days, on how many days did you do vigorous physical activities like heavy lifting, digging, aerobics, or fast bicycling?	Days per week	Adopted from Dutch version of IPAQ-SF	Reliability and validity scores based on an systematic review on the measurement properties of the original IPAQ-SF by Craig et al, 2003 (9):<br><br>Reliability: r = 0.66–0.91<br>Validity (compared to an accelerometer): r = 0.02–0.52.
6.1.1 (if 6.1=not ''no vigorous physical activities'': How much time did you usually spend doing vigorous physical activities on one of those days?	Hours/Minutes per day	Adopted from Dutch version of IPAQ-SF	
6.2: During the last 7 days, on how many days do you spend on moderate physical activities like carrying light loads, bicycling at a regular pace, or doubles tennis? Do not include walking.	Days per week	Adopted from Dutch version of IPAQ-SF	
6.2.1 (if 6.2=not ''No moderate physical activities ''): How much time do you usually spend doing moderate physical activities on one of those days?	Hours/Minutes per day	Adopted from Dutch version of IPAQ-SF	
6.3: Think about the time you spent walking in the last 7 days. This includes at work and at home, walking to travel from place to place, and any other walking that you have done solely for recreation, sport, exercise, or leisure.	Days per week	Adopted from Dutch version of IPAQ-SF	
6.3.1 (if 6.3=not ''No walking''): How much time do you usually spend walking on one of those days?	Hours/Minutes per day	Adopted from Dutch version of IPAQ-SF	
6.4: During the last 7 days, how much time did you spend sitting on a week day?	Hours/Minutes per day	Adopted from Dutch version of IPAQ-SF	
**Living environment**			
7.1.1: Does your home have an outdoor space?	Yes, garden/Yes, balcony/Yes, roof terrace/Yes, other/No	Added	No validity/reliability assessments
7.1.2 (if 7.1=not ''No"): Our outdoor space is suitable for:	3 point scale; Only calm play-All activities, including active play	Translated and adapted from EPAO	

7.2: The place where my child plays most indoors (e.g. the living room) is suitable for:	3 point scale; Only calm play-All activities, including active play	Translated and adapted from EPAO	Adaptation and Dutch translation by Gubbels et al, 2011 (5)<br><br>Test-retest reliability and validity (gold standard observer) scores of the original subscales by Ward et al, 2015 (6):<br><br>Physical activity environment Portable equipment subscale:<br>ICC = 0.93-0.96 (observer)<br>ICC = 0.59-0.74 (staff)<br>r = 0.23-0.26<br><br>Fixed equipment subscale:<br>ICC = 0.98-0.99 (observer)<br>ICC = 0.73-0.85 (staff)<br>r = 0.49-0.55<br><br>Inside space for gross motor activity subscale:<br>ICC = 0.98-0.99 (observer)<br>ICC = 0.53-0.69 (staff)<br>r = 0.02-0.06<br><br>Inter-rater reliability and validity (compared to accelerometer) scores of the original subscales by Vaughn et al, 2017 (10):
7.3: Which of the following play equipment do you have available for your child at home inside?<br><br>And<br><br>Which of the following play equipment do you have available for your child at home outside?	1) Ball or other toy to throw or roll; 2) Football goal, basketball hoop, etc.; 3) Loose toys for jumping, e.g. Skippy ball; 4) Play mat or other floor covering for playing on the floor; 5) Walking car, balance bike, tricycle or other toys to ride on; 6) Rocking horse or other materials to bounce on; 7) Sandpit and/or water toys (e.g. shovels, buckets); 8) Climbing equipment (e.g. climbing frame or slide); 9) Swing; 10) Possibility to play music or musical instruments; 11) Other play equipment	Translated and adapted from the Dutch version of EPAO	

			Indoor play equipment subscale:<br>ICC = 0.81<br>r = 0.11<br><br>Outdoor play environment subscale:<br>ICC = 0.84<br>r = 0.04
7.4: Is there a park in your neighborhood?	Yes/No/I don't know	Added, based on a questionnaire used in the Dutch 'healthy neighborhoods project'	No validity/reliability assessments
7.4.1: How satisfied are you with this park?	Report mark between 1 and 10		
7.4.2: Have you visited this park with your child in the past month?	Yes/No		
7.5: Is there a public garden in your neighborhood?	Yes/No/I don't know		
7.5.1: How satisfied are you with this public garden?	Report mark between 1 and 10		
7.5.2: Have you visited this public garden with your child in the past month?	Yes/No		
7.6: Is there a lawn in your neighborhood?	Yes/No/I don't know		
7.6.1: How satisfied are you with this lawn?	Report mark between 1 and 10		
7.6.2: Have you visited this lawn with your child in the past month?	Yes/No		
7.7: Is there a forest in your neighborhood?	Yes/No/I don't know		
7.7.1: How satisfied are you with this forest?	Report mark between 1 and 10		
7.7.2: Have you visited this forest with your child in the past month?	Yes/No		
7.8: Is there a playground in your neighborhood?	Yes/No/I don't know		
7.8.1: How satisfied are you with this playground?	Report mark between 1 and 10		
7.8.2: Have you visited this playground with your child in the past month?	Yes/No		
7.9: Is there a sports/football field in your neighborhood?	Yes/No/I don't know		

7.9.1: How satisfied are you with this sports/football field?	Report mark between 1 and 10		
7.9.2: Have you visited this sports/football field with your child in the past month?	Yes/No		
7.10: Is there another green facility in your neighborhood?'	Yes/No/I don't know		
7.10.1: How satisfied are you with this sports/football field?	Report mark between 1 and 10		
7.10.2: Have you visited the sports/football field with your child in the past month?	Yes/No		
8.1: Most streets in my neighborhood have sidewalks	10-point scale; Disagree-Agree	Adapted from the Dutch translation of the European environmental questionnaire ALPHA	Test-retest reliability scores based on Dutch, French, English and German versions of ALPHA by Spittaels et al, 2010 (11):<br><br>- item 3c<br>Agreement: 62%; ICC: 0.69<br>- item 3a:<br>Agreement: 73%; ICC: 0.74<br>- item 5c<br>Agreement: 68%; ICC: 0.58<br>- item 5d<br>Agreement: 62%; ICC: 0.61<br>- item 5e<br>Agreement: 74%; ICC: 0.57
8.2: Most streets in my neighborhood have bike lanes	10-point scale; Disagree-Agree	Adapted from the Dutch translation of the European environmental questionnaire ALPHA	
8.3: When I walk with my child in my neighborhood I feel unsafe due to traffic (for example due to heavy traffic or unsafe traffic situations)	10-point scale; Disagree-Agree	Adapted from the Dutch translation of the European environmental questionnaire ALPHA	
8.4: When I cycle with my child in my neighborhood I feel unsafe due to traffic (for example due to heavy traffic or unsafe traffic situations)	10-point scale; Disagree-Agree	Adapted from the Dutch translation of the European environmental questionnaire ALPHA	
8.5: I do not go to the playground with my child because I am afraid of crime or being harassed	10-point scale; Disagree-Agree	Adapted from the Dutch translation of the European environmental questionnaire ALPHA	

Abbreviations: BISQ-R, Brief Infant Sleep Questionnaire Revisited; CI, Confidence Interval; EPAO, Environment and Policy Assessment and Observation; ICC, Intraclass Correlation Coefficient; IIC, Inter Item Correlation; IPAQ-SF, International Physical Activity Questionnaire Short form; PPAP, Preschooler Physical Activity Parenting Practices.

[1]Sarphati Cohort questionnaire, as part of the data collection within Sarphati Cohort, where research is conducted into the growth and development of Amsterdam children; for more information, see https://www.sarphaticohort.nl/en/over-sarphati-cohort/.

[2]Based on International Standard Classification for Education (ISCED).

References

1. Gubbels JS, Sleddens EF, Raaijmakers L, Gies JM, Kremers SP. The Child-care Food and Activity Practices Questionnaire (CFAPQ): development and first validation steps. Public Health Nutr. 2016;19(11):1964-75.
2. O'Connor TM, Cerin E, Hughes SO, Robles J, Thompson DI, Mendoza JA, et al. Psychometrics of the preschooler physical activity parenting practices instrument among a Latino sample. Int J Behav Nutr Phys Act. 2014;11:3.
3. Okely T, Reilly JJ, Tremblay MS, Kariippanon KE, Draper CE, El Hamdouchi A, et al. Cross-sectional examination of 24-hour movement behaviours among 3- and 4-year-old children in urban and rural settings in low-income, middle-income and high-income countries: the SUNRISE study protocol. BMJ Open. 2021;11(10):e049267.
4. Vaughn AE, Hales D, Ward DS. Measuring the physical activity practices used by parents of preschool children. Med Sci Sports Exerc. 2013;45(12):2369-77.
5. Gubbels JS, Kremers SP, van Kann DH, Stafleu A, Candel MJ, Dagnelie PC, et al. Interaction between physical environment, social environment, and child characteristics in determining physical activity at child care. Health Psychol. 2011;30(1):84-90.
6. Ward DS, Mazzucca S, McWilliams C, Hales D. Use of the Environment and Policy Evaluation and Observation as a Self-Report Instrument (EPAO-SR) to measure nutrition and physical activity environments in child care settings: validity and reliability evidence. Int J Behav Nutr Phys Act. 2015;12:124.
7. Mindell JA, Gould RA, Tikotzy L, Leichman ES, Walters RM. Norm-referenced scoring system for the Brief Infant Sleep Questionnaire - Revised (BISQ-R). Sleep Med. 2019;63:106-14.
8. Cassanello P, Diez-Izquierdo A, Gorina N, Matilla-Santander N, Martinez-Sanchez JM, Balaguer A. [Adaptation and study of the measurement properties of a sleep questionnaire for infants and pre-school children]. An Pediatr (Engl Ed). 2018;89(4):230-7.
9. Craig CL, Marshall AL, Sjostrom M, Bauman AE, Booth ML, Ainsworth BE, et al. International physical activity questionnaire: 12-country reliability and validity. Med Sci Sports Exerc. 2003;35(8):1381-95.
10. Vaughn AE, Mazzucca S, Burney R, Ostbye T, Benjamin Neelon SE, Tovar A, Ward DS. Assessment of nutrition and physical activity environments in family child care homes: modification and psychometric testing of the Environment and Policy Assessment and Observation. BMC Public Health. 2017;17(1):680.
11. Spittaels H, Verloigne M, Gidlow C, Gloanec J, Titze S, Foster C, et al. Measuring physical activity-related environmental factors: reliability and predictive validity of the European environmental questionnaire ALPHA. Int J Behav Nutr Phys Act. 2010;7(1):48.
