# Supplementary material for: 24-hour movement behaviours in the early years, potential behavioural determinants and prospective associations with growth, motor and social–emotional development: the My Little Moves study protocol
Source: BMJ Open. 2024 Oct 22;14(10):e081836. doi: 10.1136/bmjopen-2023-081836 (PMC11499838; doi:10.1136/bmjopen-2023-081836)
Supplement: Supplementary file 3 [file bmjopen-2023-081836.reviewer_comments.pdf]

PEER REVIEW HISTORY

BMJ Open publishes all reviews undertaken for accepted manuscripts. Reviewers are asked to complete a checklist review form and are provided with free text boxes to elaborate on their assessment. These free text comments are reproduced below.

ARTICLE DETAILS

Title (Provisional)

24-hour movement behaviours in the early years, potential behavioural determinants and prospective associations with growth, motor and social-emotional development – the My Little Moves study protocol

Authors

Altenburg, Teatske M; Gubbels, Jessica S.; Arts, Jelle; Lettink, Annelinde; Veldman, Sanne; Verhoeff, Arnoud; Chinapaw, Mai

---

VERSION 1 - REVIEW

**Reviewer**	**1**
**Name**	**Hesketh, K**
**Affiliation**	**University of Cambridge, UKCRC Centre for Diet and Activity Research (CEDAR)**
**Date**	**08-Feb-2024**
**COI**	**No competing interests**

This is a well planned study and will provide important information to the field. Please find comments and suggestions below. In particular, more information is require about how the participants are allocated to each of the sub cohorts, and whether this has implications for the wider generalisability of each sub-cohort.

Abstract

The abstract currently reads as though the data collected is complete - is this the case? If so, please provide additional details about where the sample was recruited from, numbers of children approached, age at baseline etc. Please also state why different sub-cohorts were followed and whether there were differences between them.

Introduction

- Comprehensive.

- Second para, second line: yet only A few, or yet few

- Use of challenged in second paragraph – consider using complicated or made difficult instead.

Methods

- Please provide a more detailed explanation of how children were included/ allocated to each of the three sub-cohorts – was this done randomly, based on age or another mechanism?

- Please include comparable information about sub-cohorts for community (as per Sarphati/ ECEs)

- Pg 6, ln 57: 'de' Sarphati not 'the' ?

- Pg9, ln28 – is this an R package?

- Pg9, ln48 – you mention the EPAO for family care homes, but did you also ask the parents to fill this in for their homes? I'm not aware of this having been done before, so was this tested prior to roll out? Please provide more information here.

- For the EPAO proper, did you go into every setting where children had agree to participate? Again, some more information here would be useful.

- It would possibly be helpful to have an indicator or similar after each of the headings (e.g. PA measurement, growth etc) which of the cohorts this was conducted in.

- For the analysis section, I suggest including the future tense to make it clear that information is not contained in the current manuscript.

- Please provide more information about what you mean by hybrid model and make it clear whether you mean that both of the stated elements (i.e. individual and mean) are for each of the outcome variables (i.e. growth etc.) I presume? Also, presumably these models will be run separately for each outcome or is this a combined multivariate (i.e. multiple outcomes) model? If this could be clarified, that would be helpful please.

- The backward stepwise regression model paragraph requires more detail. In addition, this method has largely fallen out of favour, with a more cause-driven approach deemed better (e.g. https://journalofbigdata.springeropen.com/articles/10.1186/s40537-018-0143-6).

- You could provide more information about the planned analyses, to highlight which specific exposure – outcome relationships you are intending to explore, hypotheses etc.

Discussion

- Suggest this is written in the future tense to show that the evidence is not presented here (as it is a protocol paper).

VERSION 1 - AUTHOR RESPONSE

We would like to thank the editor for the opportunity to revise and resubmit our manuscript, and kindly refer to the attached file for our point-to-point reply.

**Reviewer's comments to the Author**		
This is a well planned study and will provide important information to the field. Please find comments and suggestions below. In particular, more information is require about how the participants are allocated to each of the sub cohorts, and whether this has implications for the wider generalisability of each sub-cohort.	We thank the reviewer for acknowledging the importance of our work, and providing valuable suggestions to improve our manuscript. We have responded to all comments in our point-by-point reply below.	
Abstract		
The abstract currently reads as though the data collected is complete - is this the case? If so, please provide additional details about where the sample was recruited from, numbers of children approached, age at baseline etc. Please also state why different sub-cohorts were followed and whether there were differences between them.	Recruitment and baseline data is completed, but follow-up data collection is still ongoing. To clarify this, we have now written the part on data collection in present tense, in both the abstract and main text. Additionally, we have provided the numbers and characteristics of included children in all sub-cohorts:<br><br>*"In sub-cohort 1, informed consent was obtained for 219 children of which 214 children (20.5 ± 12.4 months, 53% girls) were eligible. In sub-cohort 2, informed consent was obtained for 202 children of which 197 children (12.3 ± 13.0 months, 46.7% girls) were eligible–. In sub-cohort 3, informed consent was obtained for 87 children of which 84 children (19.1 ± 11.8 months, 43 % girls) were eligible."*	P7-8, L216-220
Introduction		
- Comprehensive.<br>- Second para, second line: yet only A few, or yet few<br>- Use of challenged in second paragraph – consider using complicated or made difficult instead.	We thank the reviewer for the compliments on our introduction, and have adjusted the sentence on line 88: "*only a few studies*".<br><br>Regarding the wording of "challenged" we prefer to keep this, as we prefer a positive focus with the indicated points	

	as challenges that we aim to overcome.	
Methods		
- Please provide a more detailed explanation of how children were included/ allocated to each of the three sub-cohorts – was this done randomly, based on age or another mechanism?<br><br>- Please include comparable information about sub-cohorts for community (as per Sarphati/ ECEs)	Thank you for these requests.<br>– We are happy to provide more information on how children were allocated to the three sub-cohorts, and have added this to the methods: *"At the start, waves of email invitations (Sarphati) and organisations (ECEC and Youth Health Care services, community organisations) were randomly allocated to sub-cohorts 1 and 2. At a later stage, allocation was targeted to balance the sample size across sub-cohorts. For sub-cohort 3, the data collection is more intensive (requiring in-person contact with the child) and time consuming (i.e., physical measurements and wearing accelerometers). Therefore, we recruited children for sub-cohort 3 through organizations that could support recruitment through face-to-face contact and offer a space for conducting the measurements, i.e. ECEC and Youth Health Care services."*<br>– We have also added information about recruitment from community organisations and public spaces: *"Parents and children approached via other community organisations and public spaces were recruited for participation in sub-cohort 1 or 2."*	P6, L169,176<br><br>P7, L209-210
Pg 6, ln 57: 'de' Sarphati not 'the' J	We have corrected this typo.	

Pg9, ln28 – is this an R package?	This is indeed an R package, we have added this information:<br>*"… R package GGIR version 2.9-0, …"*	P9, L284
Pg9, ln48 – you mention the EPAO for family care homes, but did you also ask the parents to fill this in for their homes? I'm not aware of this having been done before, so was this tested prior to roll out? Please provide more information here.<br>For the EPAO proper, did you go into every setting where children had agree to participate? Again, some more information here would be useful.	The EPAO was only applied to the physical and policy environment of ECEC centres, not the family home. We asked approval from managers of ECEC services to assess the physical and policy environment of the day-care/preschool, using the EPAO instrument. We did this for every setting from which children were included in the study.<br>We have reworded this sentence to clarify this:<br>*"For all children included in sub-cohort 3, recruited via ECEC services, we approached the managers for approval to assess the ECEC physical and policy environment. After approval, one research assistant assessed the ECEC environment and policy during a day long visit, using a Dutch translation of the Environment and Policy Assessment and Observation (EPAO) instrument."*	P10, L311-315
It would possibly be helpful to have an indicator or similar after each of the headings (e.g. PA measurement, growth etc) which of the cohorts this was conducted in.	We thank the reviewer for this suggestion and have added a sub-cohort indication for each of the sub-headings in the methods section.	
For the analysis section, I suggest including the future tense to make it clear that information is not contained in the current manuscript.	We agree with the reviewer that the future tense would clarify that the analyses have not yet been conducted. As the recruitment and baseline data collection is finished, we have now written the procedures regarding recruitment in the past tense, and the data collection procedures in present tense. We believe that switching between present, past and future tense	

	would not benefit the readability of the manuscript, thus we prefer to keep the discussion in the present tense.	
Please provide more information about what you mean by hybrid model and make it clear whether you mean that both of the stated elements (i.e. individual and mean) are for each of the outcome variables (i.e. growth etc.) I presume? Also, presumably these models will be run separately for each outcome or is this a combined multivariate (i.e. multiple outcomes) model? If this could be clarified, that would be helpful please.	We apologize for not being clear about the hybrid mixed model analysis and whether models will be run separately for all outcomes. We have clarified this paragraph as follows:<br><br>*"Hybrid mixed model analyses are used to assess the prospective associations of 24-hour movement behaviours (based on summary statistics of the My Little Moves app data and/or accelerometer data) with young children's growth, motor and social-emotional development, respectively. Using a hybrid model, we include both the individual mean score and the deviation score around the individual mean as independent variables, which enables us to disentangle the between- and the within-subjects part of the prospective associations (42)."*	P12, L386-391
The backward stepwise regression model paragraph requires more detail. In addition, this method has largely fallen out of favour, with a more cause-driven approach deemed better (e.g. https://journalofbigdata.springeropen.com/articles/10.1186/s40537-018-0143-6).	We thank the reviewer for pointing us to this interesting paper. The more cause-driven approach is exactly what we aim to do. We have rephrased this paragraph to clarify this:<br><br>*"Potential determinants of young children's 24-hour movement behaviours (based on summary measures of the My Little Moves app data) are explored using regression analysis, including the child, parental and environmental factors as well as two-way interactions between these factors. First, 5-10 potential determinants and two-way*	P13, L400-408

	interactions between these potential determinants are selected, based on relevant literature and theories. Next, associations of the selected potential determinants and the two-way interactions with young children's 24-hour movement behaviours are explored using regression analysis."	
You could provide more information about the planned analyses, to highlight which specific exposure – outcome relationships you are intending to explore, hypotheses etc.	We have elaborated on the planned analyses, which we believe now clearly explains the analyses we plan to conduct:<br><br>*"By using the Spearman-Brown prophecy formula, inputting single-day intra-class correlation coefficients, the minimum number of days and hours of registration time required to achieve a reliability of 0.70 is determined, both for parent-reported and accelerometer data on 24-hour movement behaviours (40, 41). The construct validity of the My Little Moves app and accelerometers is examined by fitting general linear mixed-effects models to test for differences in accelerometer-derived acceleration across app-derived activity categories as well as physical activity, sedentary behaviour and sleep.*<br><br>*Hybrid mixed model analyses are used to assess the prospective associations of 24-hour movement behaviours (based on summary statistics of the My Little Moves app data and/or accelerometer data) with young children's growth, motor and social-emotional development, respectively. Using a hybrid model, we include both the individual mean*	P12-13, L374-398

	score and the deviation score around the individual mean as independent variables, which enables us to disentangle the between- and the within-subjects part of the prospective associations (42). The co-dependency of the 24-hour movement behaviours is addressed using compositional data analysis (17). All analyses are adjusted for children's age and gender.<br><br>*Additionally, latent profile analysis is applied to the My Little Moves app data to identify 24-hour movement behaviour profiles and sequence mapping is applied to accelerometer data to explore the alternation and accumulation of 24-hour movement behaviours (18).*	
Discussion		
Suggest this is written in the future tense to show that the evidence is not presented here (as it is a protocol paper).	We kindly refer to our response to one of the previous comments of the reviewer: we believe switching between present, past and future tense does not benefit the readability of the manuscript, therefore, we prefer to keep the discussion in the present tense.	
